# Risk-prone territories for spreading tuberculosis, temporal trends and their determinants in a high burden city from São Paulo State, Brazil

**DOI:** 10.1186/s12879-022-07500-5

**Published:** 2022-06-02

**Authors:** Thaís Zamboni Berra, Antônio Carlos Vieira Ramos, Luiz Henrique Arroyo, Felipe Mendes Delpino, Juliane de Almeida Crispim, Yan Mathias Alves, Felipe Lima dos Santos, Fernanda Bruzadelli Paulino da Costa, Márcio Souza dos Santos, Luana Seles Alves, Regina Célia Fiorati, Aline Aparecida Monroe, Dulce Gomes, Ricardo Alexandre Arcêncio

**Affiliations:** 1 Department of Maternal-Infant and Public Health Nursing Graduate Program, University of São Paulo at Ribeirão Preto College of Nursing, Avenida dos Bandeirantes, 3900, Monte Alegre, Ribeirão Preto, São Paulo, 14040-902 Brazil; 2 Department of Neurosciences and Behavioral Sciences, Faculty of Medicine, University of São Paulo at Ribeirão Preto, Ribeirão Preto, São Paulo, Brazil; 3 Mathematics Department, University of Évora, Rua Romão Ramalho, 59, Évora, Portugal

**Keywords:** Tuberculosis, Spatial analysis, Temporal trend

## Abstract

**Objectives:**

To identify risk-prone areas for the spread of tuberculosis, analyze spatial variation and temporal trends of the disease in these areas and identify their determinants in a high burden city.

**Methods:**

An ecological study was carried out in Ribeirão Preto, São Paulo, Brazil. The population was composed of pulmonary tuberculosis cases reported in the Tuberculosis Patient Control System between 2006 and 2017. Seasonal Trend Decomposition using the Loess decomposition method was used. Spatial and spatiotemporal scanning statistics were applied to identify risk areas. Spatial Variation in Temporal Trends (SVTT) was used to detect risk-prone territories with changes in the temporal trend. Finally, Pearson's Chi-square test was performed to identify factors associated with the epidemiological situation in the municipality.

**Results:**

Between 2006 and 2017, 1760 cases of pulmonary tuberculosis were reported in the municipality. With spatial scanning, four groups of clusters were identified with relative risks (RR) from 0.19 to 0.52, 1.73, 2.07, and 2.68 to 2.72. With the space–time scan, four clusters were also identified with RR of 0.13 (2008–2013), 1.94 (2010–2015), 2.34 (2006 to 2011), and 2.84 (2014–2017). With the SVTT, a cluster was identified with RR 0.11, an internal time trend of growth (+ 0.09%/year), and an external time trend of decrease (− 0.06%/year). Finally, three risk factors and three protective factors that are associated with the epidemiological situation in the municipality were identified, being: race/brown color (OR: 1.26), without education (OR: 1.71), retired (OR: 1.35), 15 years or more of study (OR: 0.73), not having HIV (OR: 0.55) and not having diabetes (OR: 0.35).

**Conclusion:**

The importance of using spatial analysis tools in identifying areas that should be prioritized for TB control is highlighted, and greater attention is necessary for individuals who fit the profile indicated as “at risk” for the disease.

**Supplementary Information:**

The online version contains supplementary material available at 10.1186/s12879-022-07500-5.

## Background

Tuberculosis is one of the oldest diseases in the world and is still the cause of illness for millions of people each year. It is estimated that in 2020 there were approximately 10 million cases of tuberculosis in the world, with 56% of these cases affecting males, 33% females, and 11% children under 15 years of age [1]. In addition to these alarming statistics, the WHO still estimates 1.3 million deaths from tuberculosis and more than 214,000 deaths caused by co-infection with tuberculosis and human immunodeficiency virus (TB-HIV) [1].

Brazil remains among the 30 countries with a high burden for TB and TB-HIV co-infection, being considered a priority for disease control globally by the WHO [2]. In 2020, Brazil recorded about 67,000 new TB cases, presenting an incidence coefficient of 31.6 cases per 100 thousand inhabitants, also, in 2019, about 4500 deaths from the disease, was presented with a coefficient of mortality of 2.2 deaths per 100 thousand inhabitants [2].

It is known that the incidence of any disease changes over time and that the time trend also varies according to the geographic region. Thus, the importance of monitoring the emerging spatial patterns and temporal risk trends for tuberculosis is emphasized to provide additional information which helps prevent the disease, implement control measures and address new health risks within a geographical region [3].

In scientific literature, many studies have analyzed the behavior of the disease in a territory under study and sought to identify areas of risk. However, few studies have addressed the temporal behavior of tuberculosis and concomitantly placed variations in the temporal trends in each territory at risk. Therefore, this study aimed to identify risk-prone areas for the spread of tuberculosis, analyze spatial variation and temporal trends in these areas, and their determinants in a municipality with high endemicity due to the disease.

## Methods

### Research design and scenario

An ecological study [4] was carried out in Ribeirão Preto, a city in the interior of the state of São Paulo (SP)—Brazil. Located 314 km from the capital, Ribeirão Preto has an area of approximately 650 km^2^ and a high population density of 995.3 inhabitants/km^2^. It also had an estimated population of 711,825 inhabitants in 2020, of which 99.7% live in urban areas [5].

The unit of ecological analysis used in the study is the census sector. Ribeirão Preto is composed of 972 census sectors, of which 956 were considered in the present study because they are urban areas of the municipality.

### Population

The study population was composed of cases of pulmonary tuberculosis reported through the Tuberculosis Control System (TBWeb) [6] from 2006 to 2017. Data were obtained from the Municipal Tuberculosis Control Program of the Ribeirão Preto Municipal Secretariat. Only pulmonary tuberculosis cases were selected since it is the unique contagious form of the disease and with chances of spreading in the territory.

It was adopted as a selection criterion that the notification was carried out between 2006 and 2017, with only one registration per person, the most current registration being selected if there was more than one entry in the system and only residents in urban area of the city of Ribeirão Preto were included. It is noteworthy that only pulmonary tuberculosis records were considered so that extrapulmonary or concomitant forms (pulmonary and extrapulmonary forms together) were excluded as part of the exclusion criteria.

### Analysis plan

#### Time series analysis

Initially, monthly time series of tuberculosis cases were constructed, spanning between January 2006 to December 2017. To verify the behavior of the time series over the study period and also its trend, the decomposition method called Seasonal Trend Decomposition using Loess (STL) was used, based on a locally weighted regression [7]. This analysis was performed using RStudio software through the forecast package [8].

#### Identification of clusters

The georeferencing of pulmonary tuberculosis cases were performed using the Google Earth Pro^®^ software in order to obtain the geographical coordinates (latitude and longitude) of the residential addresses of the notified cases.

In order to identify areas at higher risk for pulmonary tuberculosis, the spatial analysis technique called scanning statistics, developed by Kulldorff and Nagarwalla [9] was used.

It is considered as a null hypothesis if there is no high or low-risk cluster, which means, the entire population has the same probability of contracting pulmonary tuberculosis, regardless of its location; while the alternative hypothesis predicts the existence of clusters that are areas in which the population would be more or less likely to contract the disease [10].

Unlike the purely spatial scan that is based on circles, in the space–time scan, cylinders are created around each centroid, incorporating time as a variable of interest, it is possible to verify the existence of clusters in a given area, and also prove that in a specific period of time, there was a greater or lesser proportion of cases when compared to the other areas that were analyzed [11].

Still referring to the analysis of cluster detection, the SVTT technique was also performed, which differs from the other analyses presented by calculating the temporal trend of the clusters [3]. The time trend is calculated inside and outside the scan circle, we call the internal temporal trend (ITT) the change in the time trend of the event within a cluster and the external temporal trend (ETT) is called the trend of all other areas that do not belong to the cluster in question. Therefore, what is statistically significant in this analysis are the temporal trends and not the cluster formation as in spatial and space–time scanning [3, 11].

SVTT is considered a null hypothesis when there is no difference in the temporal trends in the analyzed areas while we have as an alternative hypothesis the occurrence that the temporal trends are statistically different.

In addition, the relative risk (RR) and 95% confidence interval (95%CI) of each cluster was calculated, allowing the comparison of information in different areas, with the exception of SVTT, because, as explained above, it is emphasized that what is significant in this analysis are ITT and ETT, so that the RR of the identified cluster may not be within the CI. Clusters with p < 0.05 were considered statistically significant. The analyses were performed using SaTScan software 9.3, and thematic maps were created using ArcGis software 10.5.

#### Descriptive and association analysis

In order to identify factors associated with the epidemiological situation in the municipality, an exploratory analysis was carried out (absolute and relative frequencies), and then the association of these variables with the fact of living in a risk area was identified by means of Pearson's chi-square analysis (χ^2^) and for the variables that were statistically significant (p < 0.05), the Odds Ratio (OR) and 95%CI were calculated using the IBM SPSS version 25 software.

We emphasize that the χ^2^ test will be used to identify whether there is an association between the variables and the OR to quantify this association and classify whether it characterizes risk or protection.

We also emphasize that the dichotomized dependent variable was having tuberculosis and residing in an identified cluster (0 and 1) and this variable was crossed with all the independent variables that were also dichotomized (0 and 1) and that category analyzed was considered as a reference. Only for variables with two categories (HIV, diabetes, alcoholism, mental disease, drug addiction and smoking) we adopted as a reference category not having the comorbidity or not using substances.

By observing the association between two independent dichotomous categorical variables, the Chi-square test can be used to generate a traditional p-value that verifies the existence or not of association. However, within the applied statistics, the chi-square p-value is of little value due to the loss of precision, accuracy and variance that comes with categorical variables and, in this way, the use of the Odds Ratio calculation is indicated with a confidence interval of 95% for a more accurate measurement.

Additionally, a binary logistic regression was also conducted because it is a more robust analysis. Initially, from the variables present in the TBWeb notification form, the variables that could explain the variable of interest were chosen. All selected variables were dichotomized (0 and 1) and the dependent variable (having TB and residing in the risk cluster) was also dichotomized.

Then, the selected variables were tested for multicollinearity based on the variance inflation factor (VIF), and those with an index greater than ten were excluded [12] (Additional file 1). After the selection process of the independent variables was completed, logistic regression was conducted using the RStudio 4.0.4 program.

The backward selection was used to insert the variables in the model. This method incorporates all the variables in the model and one by one it is removed and the individual contributions of the variables to this model are investigated, and the worst performing variable is eliminated, comparing the complete model with the reduced model, by removing such variable. After exhausting all the possibilities of the analysis, the best model was chosen based on the lowest values of the Akaike Information Criterion (AIC) [13]. It is also noteworthy that for the final model with the best comparison parameter, the Odds Ratio (OR) and their respective 95%CI were calculated.

## Results

Between 2006 and 2017, 2,259 tuberculosis cases were reported in Ribeirão Preto, of which 1760 (77.9%) were pulmonary tuberculosis. The minimum age of the cases was two months, and the maximum was 102 years old, with an average of 42 years old and a standard deviation of 17.3.

**Fig. 1:**
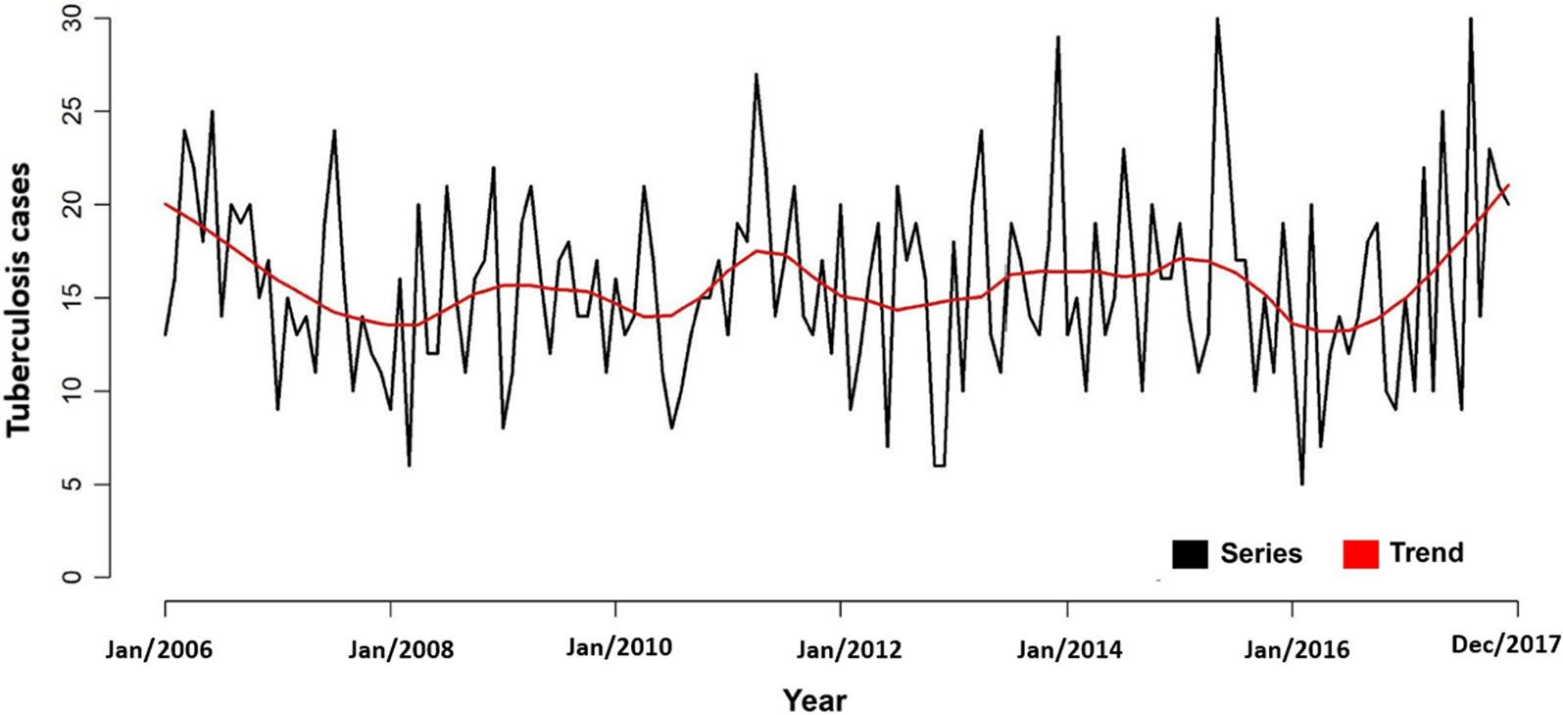
Series and time trend of pulmonary tuberculosis cases, Ribeirão Preto-SP, Brazil (2006–2017)

Figure 1 shows the behavior of the pulmonary tuberculosis time series and its time trend over the study period. It is possible to observe the presence of peaks and falls in specific periods of the years.

In the spatial analysis stage, 112 cases were excluded because they lived in a rural area, the address field on the notification form was not valid (wrong, blank, incomplete address or address of government agencies—hospitals, health units, prisons), making georeferencing impossible. Therefore, 1648 cases (93.6%) were georeferenced and integrated into the following analyses.

**Fig. 2:**
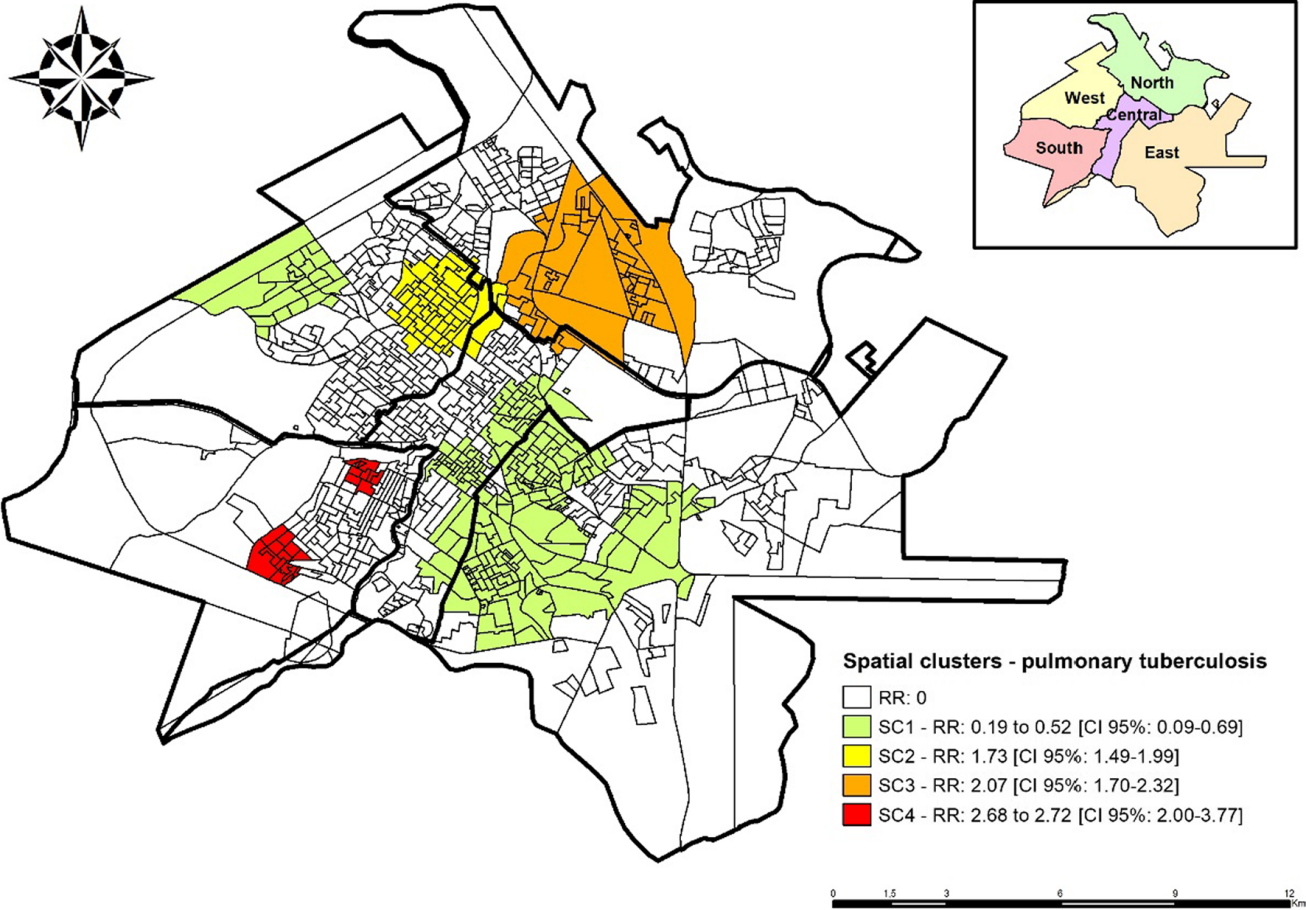
Areas of spatial risk for the occurrence of pulmonary tuberculosis, Ribeirão Preto, SP, Brazil (2006–2017)

With the application of the purely spatial scanning technique, it was possible to identify four groups of statistically significant clusters (p < 0.01) (Fig. 2). Spatial cluster 1 (SC1), considered as protection for the event, presented RR: 0.19–0.52 (95% CI: 0.09–0.69), composed of 238 census sectors in the eastern, western, and central districts of the municipality. Spatial cluster 2 (SC2) with RR: 1.73 (95%CI: 1.49–1.99) composed 60 census sectors in the west district, two census sectors in the central district, and two census sectors in the north district.

Spatial cluster 3 (SC3), presented RR: 2.07 (95%CI: 1.70–2.32), was composed of 52 census sectors in the north district and two census sectors in the central district, and finally, spatial cluster 4 (SC4) with RR: 2.68 to 2.72 (95%CI: 2.00–3.77), was composed of 24 census sectors in the southern district.

**Fig. 3:**
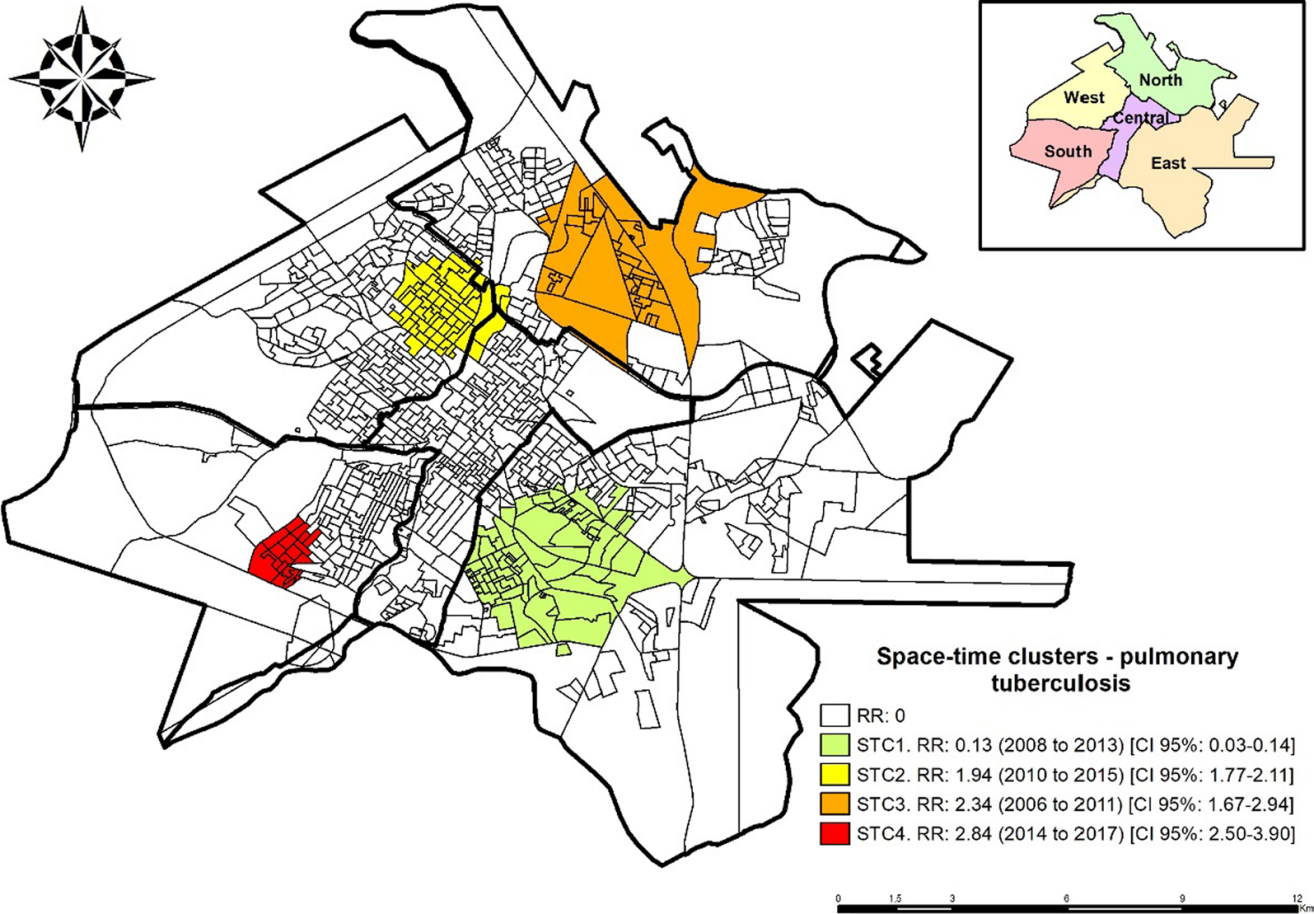
Space–time risk areas for the occurrence of pulmonary tuberculosis, Ribeirão Preto, SP, Brazil (2006–2017)

With the space–time scan, it was possible to identify four statistically significant clusters (p < 0.01), one being protective and three at risk for pulmonary tuberculosis (Fig. 3).

The space–time cluster 1 (STC1), considered protection for the event, presented RR: 0.13 (95%CI: 0.03–0.26) between 2008 and 2013, was composed of 87 census sectors in the eastern district. The space–time cluster 2 (STC2) presented RR: 1.94 (95%CI: 1.77–2.11) between 2010 and 2015; it was composed of 58 census sectors in the west, north, and central district.

The space–time cluster 3 (STC3), with RR: 2.34 (95%CI: 1.67–2.94) and the period from 2006 to 2011, was composed of 44 census sectors in the northern district and the space–time cluster 4 (STC4) (p < 0.01) presented RR: 2.84 (95%CI: 2.50–3.90) between the years 2014 and 2017, it was composed of 20 census sectors in the southern district.

**Fig. 4:**
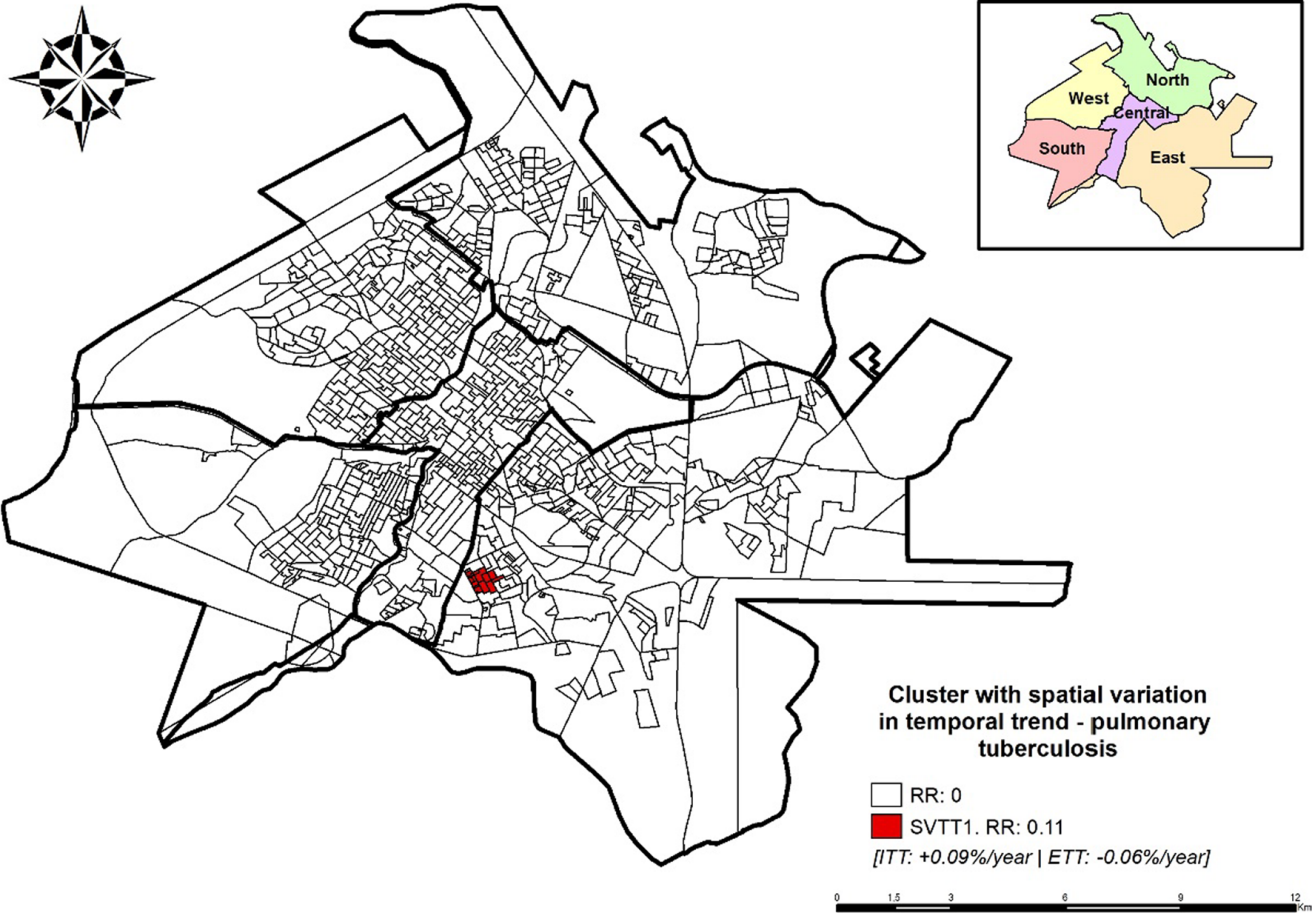
Areas with spatial variation in temporal trends for the occurrence of pulmonary tuberculosis, Ribeirão Preto, SP, Brazil (2006–2017)

With the application of SVTT, it was found that the cases of pulmonary tuberculosis in Ribeirão Preto showed an average decrease of 0.12% per year, making the findings presented in Fig. 1 in which periods of peaks and falls were more evident. Still, with this analysis, it was possible to identify a cluster with a statistically significant variation in the temporal trend (p < 0.01) (Fig. 4).

The cluster with spatial variation in temporal trends 1 (SVTT1) has RR: 0.11 and was composed of 13 census sectors in the eastern district of the municipality. The cluster, classified as protection for the event, presented an ITT of growth (+ 0.09%/year), while the ETT indicated a decrease (–0.06%/year).

Table 1 shows the clinical-epidemiological profile of all pulmonary tuberculosis cases reported in Ribeirão Preto from 2006 to 2017 and the clinical-epidemiological profile of patients of pulmonary tuberculosis resident in the clusters identified with the techniques of the scan applied.

**Table 1:** Clinical and epidemiological profile of pulmonary tuberculosis cases, Ribeirão Preto, SP, Brazil (2006–2017)

Variables	All reported cases N (1760) (%)	Cases residing in clusters N (680) (%)	Pearson’s Chi-square χ^2^ (p-value)	Odds ratio (95%CI)
Age				
0–14 years	74 (4.2%)	32 (4.7)	0.69 (0.41)	*NA*
15–59 years	1393 (79.1)	541 (79.6)	0.11 (0.73)	*NA*
60 years or older	277 (15.7)	100 (14.7)	0.89 (0.34)	*NA*
Ignored	16 (0.9)	7 (1.0)	*NA*	*NA*
Sex				
Male	1229 (69.8)	468 (68.8)	0.53 (0.47)	*NA*
Female	531 (30.2)	212 (31.2)	*NA*	*NA*
Race				
Yellow	2 (0.1)	*NA*	1.26 (0,26)	*NA*
White	704 (40.0)	267 (39.3)	0.25 (0,62)	*NA*
Brown	331 (18.8)	143 (21.0)	3.58 (0,05)	1.77 (1.01–1.86)
Black	141 (8.0)	50 (7.4)	0.65 (0,42)	*NA*
Ignored	582 (33.0)	65 (9.6)	*NA*	*NA*
Years of study				
No study	80 (4.5)	36 (5.3)	1.43 (0.23)	*1.16 (1.03–1.85)*
1–3 years	160 (9.1)	65 (9.6)	0.29 (0.59)	*NA*
4–7 years	500 (28.4)	198 (29.1)	0.27 (0.60)	*NA*
8–11 years	241 (13.7)	77 (11.3)	5.26 (0.06)	NA
12–14 years	51 (2.9)	15 (2.2)	1.88 (0.17)	*NA*
15 years or more	29 (1.6)	17 (2.5)	4.96 (0.02)	0.73 (0.55–0.98)
HIV				
Positive	313 (17.8)	117 (17.2)	*NA*	*NA*
Negative	1447 (82.2)	563 (82.8)	4.38 (0.03)	0.55 (0.08–0.63)
Diabetes				
Yes	84 (4.8)	39 (5.7)	*NA*	*NA*
No	1676 (95.2)	641 (94.3)	2.25 (0.03)	0.35 (0.09–0.73)
Alcoholism				
Yes	365 (20.7)	144 (21.2)	*NA*	*NA*
No	1395 (79.3)	536 (78.8)	0.12 (0.71)	*NA*
Mental disease				
Yes	34 (1.9)	18 (2.6)	*NA*	*NA*
No	1726 (98.1)	662 (97.4)	2.99 (0.08)	*NA*
Drug addiction				
Yes	209 (11.9)	91 (13.4)	*NA*	*NA*
No	1551 (88.1)	589 (86.6)	2.40 (0.12)	*NA*
Smoking				
Yes	123 (7.0)	52 (7.6)	*NA*	*NA*
No	1637 (93.0)	628 (92.4)	0.73 (0.39)	*NA*

From the analysis of Pearson's chi-square analysis (χ^2^) and OR, it was possible to identify two risk factors and three protection factors for the analyzed event. In the identified areas, mixed-race people (brown) are 1.77 times more likely and people without any studies are 1.16 times more likely to get sick from pulmonary tuberculosis than people living in other areas of the municipality.

Higher education (15 years or more of study) was identified as a protective factor for tuberculosis in the identified areas (OR: 0.73). Also classified as protection against the event are HIV-negative people who do not have diabetes (OR: 0.55 and 0.35, respectively).

From the logistic regression analysis, the variables that showed multicollinearity (VIF value > 10) were excluded (Supplementary material), as follows: age (15–59 years and 60 years or older) and sex, the other variables entered the logistic regression stage and the final model was composed of nine variables. Through OR, it was possible to identify three risk factors and two protective factors associated with the epidemiological situation in the municipality (Table 2). In the identified areas, people of race/color brown are 1.26 times more likely to fall ill with pulmonary tuberculosis and also people without any education (OR: 1.71).

**Table 2:** Factors associated with the epidemiological situation in Ribeirão Preto, SP, Brazil (2006–2017)

Variable	Coefficient (p-value)	Odds ratio (CI95%)
Race/color brown	0.23 (0.05)	1.26 (1.10–1.60)
Education: no study	1.26 (0.02)	1.71 (1.53–1.95)
Education: 8–11 years of study	0.32 (0.03)	0.72 (0.53–0.96)
Education: 12–14 years of study	− 0.45 (0.14)	0.63 (0.33–1.14)
Education: + 15 years of study	0.81 (0.03)	0.26 (0.07–0.91)
Diabetes: no	− 0.33 (0.13)	0.71 (0.46–1.11)
Mental illness: no	− 0.54 (0.11)	0.57 (0.28–1.15)
Use of illicit drugs: no	− 0.23 (0.11)	0.79 (0.58–1.06)

Higher education (8–11 years of study and 15 years or more of study) was identified as a protective factor against tuberculosis in the identified areas (OR: 0.72 and 0.26, respectively).

## Discussion

The study has evidenced the risk-prone territories for TB spread and the changes in the clusters over the years. When analyzing the time series of pulmonary tuberculosis cases between 2006 and 2017, it was possible to observe a series of undulations in its temporal trend, which was later classified through the SVTT, which indicated an average decrease of 0.12% per year of pulmonary tuberculosis in Ribeirão Preto, SP, Brazil.

With the analysis of purely spatial scanning, it was possible to verify the formation of clusters in areas that can be considered at risk for the occurrence and transmissibility of pulmonary tuberculosis.

SC4, classified with the highest RR (2.68–2.72) (95%CI: 2.00–3.77), was located in the southern district where the largest subnormal agglomerate (slums) of residents is found in the municipality, and it is noteworthy that Family Health Units have not yet been implemented in this area [14, 15]. SC3 (RR: 2.07) (95%CI: 1.70–2.32), located mostly in the northern district, is the region with the highest population density in the municipality and concentrates the largest number of residents per residence. It is worthy of note that there is a settlement of rural workers in this district, and this region also concentrates the most significant number of subnormal agglomerates (slums) in the city [15].

The SC2 (RR: 1.73) (95%CI: 1.49–1.99) identified in the western district of the municipality has one of the lowest municipal human development rates in the city, with the population receiving mostly two minimum wages. It is the region with the largest number of health units and has the highest percentage of exclusive users of the Unified Health System (SUS) in the city [15].

Finally, SC1 was classified as protection for the event (RR: 0.19–0.52) (95% CI: 0.09–0.69) and located in the eastern, western, and central districts. It is noteworthy that the center of Ribeirão Preto has the lowest density of inhabitants per residence and presents mainly commercial properties. In the eastern community, the population has the highest municipal income and the highest education level. It is noteworthy that this region has areas of expansion of high-standard condominiums. However, there are also population clusters here with lower socioeconomic resources that deserve attention because although this population is located in a more economically favored region in the municipality, they do not have the same resources and there is risk for other people who live in this area and thus can cause outbreak of tuberculosis or other infectious diseases [14, 15].

With the use of the space–time scanning statistics, four statistically significant clusters were identified; one for protection against illness from pulmonary tuberculosis (STC1; RR: 0.13) (95%CI: 0.03–0.26) between the years 2008 and 2013 in the eastern district, which may indicate that during this period there were improvements in surveillance services in health, such as awareness campaigns and active search for respiratory symptoms or probably there were fewer diagnoses of the disease in this period or no notification of new cases.

In contrast, STC2 (RR: 1.94) (95%CI: 1.77–2.11) in the period from 2010 to 2015, mainly in the western region, and STC3 (RR: 2.34) (95%CI: 1.67–2.94) in the period from 2006 to 2011 in the north district were classified as risk, which may indicate that there was intense activity of active search for respiratory symptoms, and therefore, there were more notifications than other regions of the municipality.

STC4 (RR: 2.84) (95%CI: 2.50–3.90) was identified in the southern district between the years 2014 to 2017. In 2014, the diagnosis was implemented in the municipality of Ribeirão Preto using the rapid molecular test for tuberculosis (RMT-TB) performed through GeneXpert^®^MTB/RIF. The system is commonly used for the detection of *Mycobacterium tuberculosis* and resistance to rifampicin (the primary drug used in the treatment) automatically [16, 17].

The municipality has two rapid diagnostic devices, one in the west and the other in the south, and studies [16–19] carried out to compare the RMT-TB with the tests commonly performed showed that the sensitivity of the RMT-TB for a sputum sample was 72.5% (for three samples it reached 90%) and the specificity reached 99%. Therefore, the hypothesis is raised that the high number of cases diagnosed in this region may be related to the implementation of RMT-TB in the municipality.

Through the use of the SVTT, a cluster with RR: 0.11 (event protection) was identified in the eastern region of the municipality, which, in the analysis of purely spatial scanning statistics, was identified as protection for the event (RR: 0.19–0.52). The SVTT1 cluster indicated a specific area in the east with growth ITT (0.09%/year), while outside that area, the identified time trend is decreasing (0.06%/year).

It is important that municipal managers focus on strategies to combat tuberculosis in this region with the identification of respiratory symptoms and their communicants. In addition, aiming at improving the epidemiological indicators of tuberculosis, a very necessary and effective tool is the periodic training of health professionals not only in the biological sense but also to understand the epidemiological and social reality of the territory in which they are inserted, so that they can understand the relationship between tuberculosis and the social determinants of health in order to fully look at the individual [20].

Geography and its sub-areas of knowledge, including geoprocessing, have great power to support decision-making based on the analysis of cases of a given disease, uniting their socio-spatial characteristics so that it is possible to highlight the disparities between population groups and also the delimitation of risk and/or spatial protection areas for a given event of interest.

Over the years with the evolution of technology, several studies have sought to understand the behavior of tuberculosis in space in Brazil [21–24], but few studies used temporal [25, 26] or Spatio-temporal [27, 28] approaches, and the numbers of studies that combined both techniques are even smaller [29, 30]. Therefore, we encourage new studies to be carried out using spatial and temporal processes.

In the scientific literature, there are a large number of studies that have analyzed the behavior of the disease in the territory, and that sought to identify areas of risk. However, few studies have addressed the temporal behavior of tuberculosis or sought to identify variations in the temporal trends in the risk territories.

With the association analysis, it was identified that, in the risk areas classified in the municipality, people of brown race/color were 1.26 (95%CI: 1.10–1.60) times more likely to get sick with pulmonary tuberculosis, corroborating the findings of the χ^2^ analysis, which indicated OR = 1.77 (95%CI: 1.01–1.86) for this population. Other studies have shown that people of mixed race/color are less likely to be cured [31] and that the mortality rate from tuberculosis is growing in this population [32].

No plausible biological justification or relationship was found in the literature to justify this difference between brown race/color and other classifications, but the construction of Brazilian Society [33] and the social determinants of health [34] must be taken into account.

It was also identified that people without any education had 1.71 (95%CI: 1.53–1.95) times more chance, and people with high schooling (8–11 years of study and 15 years or more of study) have less chance (OR: 0.72 (95%CI: 0.53–0.96) and 0.26 (95%CI: 0.07–0.91), respectively) of falling ill with pulmonary tuberculosis in high-risk areas in the municipality, corroborating the results of the χ^2^ analysis, which indicated OR = 1.16 (95%CI: 1.03–1.85) for people without schooling and OR = 0.73 (95%CI: 0.55–0.98) for people with 15 years of schooling or more. In a literature review[35] conducted with the aim of relating the level of education to infection with *Mycobacterium tuberculosis*, it was identified that the educational level of people with tuberculosis is directly related to income, highlighting the relationship between tuberculosis and social conditions of life.

Additionally, the χ^2^ analysis also identified not having HIV or diabetes as protection for the event (OR: 0.55 (95%CI: 0.08–0.63) and 0.35 (95%CI: 0.09–0.73), respectively). It is known that HIV is an aggressive virus that attacks the immune system, and, in this way, the chances of contracting opportunistic infections such as tuberculosis increase significantly. The impact of tuberculosis co-infection with HIV in the body is bidirectional; as the HIV virus grows in the body, the risk and progression of other infections, such as tuberculosis, also increases. In this way, tuberculosis delays the recovery of CD4 T-cells that are also destroyed by HIV, which increases the progression of the disease to AIDS and, consequently, the deaths from tuberculosis in PLHIV [36–38].

The same is true for people with TB and diabetes, so TB makes glycemic control difficult, and in turn, high blood glucose makes TB worse [39, 40]. It was identified in studies [39, 40] that people with diabetes had a 2.44–8.33 times greater chance of developing tuberculosis than those without the disease. This is because people with diabetes have decreased cellular and humoral immunity, in addition to hyperglycemia and cellular insulinopenia, which have effects on the function of macrophages and lymphocytes, thus increasing the chance of infections [41–43].

Concerning the limitations of this study, it was an ecological study; the so-called ecological fallacy stands out in that because variables are used at the aggregate level, the results may not represent associations at the individual level. It is also worth mentioning the use of secondary data sources, which may include incomplete data or typos.

We would also like to emphasize the importance and also encourage further studies using different methodologies to further explore tuberculosis and also its latent and extrapulmonary forms.

## Conclusions

In view of the above, using statistical techniques and spatial analysis to understand the behavior of the disease over time and the location of areas in the municipality in which tuberculosis is a problem and using the characteristics of its surroundings to estimate the population at risk can assist managers in making assertive decisions, so that it becomes easier to understand the process about the chain of transmission of the disease and the entire context in which that population is inserted.

## Supplementary Information

**Additional file 1: Table S1. **Test for multicollinearity based on the variance inflation factor (VIF).

## Data Availability

The data that support the findings of this study are available from the Municipal Health Secretariat of Ribeirão Preto, but restrictions were applied to the availability of these data used under license for the current study, and so are not publicly available. Data are, however, available from the authors upon reasonable request and with permission of the Municipal Health Secretariat of Ribeirão Preto.

## Abbreviations

95%CI: 95% Confidence interval
ETT: External temporal trend
HIV: Human immunodeficiency virus
IBGE: Institute of Geography and Statistics
ITT: Internal temporal trend
Km: Kilometers
Km^2^: Square kilometers
OR: Odds ratio
PLHIV: People living with the human immunodeficiency virus
RMT-TB: Rapid molecular test for tuberculosis
RR: Relative risk
SC1: Spatial cluster 1
SC2: Spatial cluster 2
SC3: Spatial cluster 3
SC4: Spatial cluster 4
SINAN: Information System for Notifiable Diseases
SP: São Paulo
STC1: Space–time cluster 1
STC2: Space–time cluster 2
STC3: Space–time cluster 3
STC4: Space–time cluster 4
STL: Seasonal Trend Decomposition using Loess
SUS: Unified Health System
SVTT: Spatial variation in temporal trends
SVTT1: Cluster with spatial variation in temporal trends 1
TB-HIV: Co-infection with tuberculosis and human immunodeficiency virus
TBWeb: Tuberculosis Control system
WHO: World Health Organization
