# Supplementary material for: Risk-prone territories for spreading tuberculosis, temporal trends and their determinants in a high burden city from São Paulo State, Brazil
Source: BMC Infect Dis. 2022 Jun 2;22:515. doi: 10.1186/s12879-022-07500-5 (PMC9161466; doi:10.1186/s12879-022-07500-5)
Supplement: Supplementary file 1 — Additional file 1: Table S1. Test for multicollinearity based on the variance inflation factor (VIF). [file 12879_2022_7500_MOESM1_ESM.docx]

**Supplementary table: Test for multicollinearity based on the variance inflation factor (VIF)**

| **Variables** | **VIF** |
| --- | --- |
| **Age** | |
| 0 to 14 years | 5.44 |
| 15 to 59 years | 18.60 |
| 60 years or older | 15.64 |
| **Sex** | |
| Male | 12.11 |
| Female | - |
| **Race** | |
| Yellow | 1.01 |
| White | 1.53 |
| Brown | 1.40 |
| Black | 1.24 |
| **Years of study** | |
| No study | 1.12 |
| 1 to 3 years | 1.17 |
| 4 to 7 years | 1.40 |
| 8 to 11 years | 1.26 |
| 12 to 14 years | 1.10 |
| 15 years or more | 1.05 |
| **HIV** | |
| Positive | - |
| Negative | 1.03 |
| **Diabetes** | |
| Yes | - |
| No | 1.01 |
| **Alcoholism** | |
| Yes | - |
| No | 1.16 |
| **Mental disease** | |
| Yes | - |
| No | 1.01 |
| **Drug addiction** | |
| Yes | - |
| No | 1.13 |
| **Smoking** | |
| Yes | - |
| No | 1.11 |

*Dependent variable: cases with tuberculosis and residents in the identified clusters
